# Supplementary figures and images for: Identification of TIA1 mRNA targets during human neuronal development
Source: Mol Biol Rep. 2021 Aug 19;48(9):6349–61. doi: 10.1007/s11033-021-06634-0 (PMC8437838; doi:10.1007/s11033-021-06634-0)

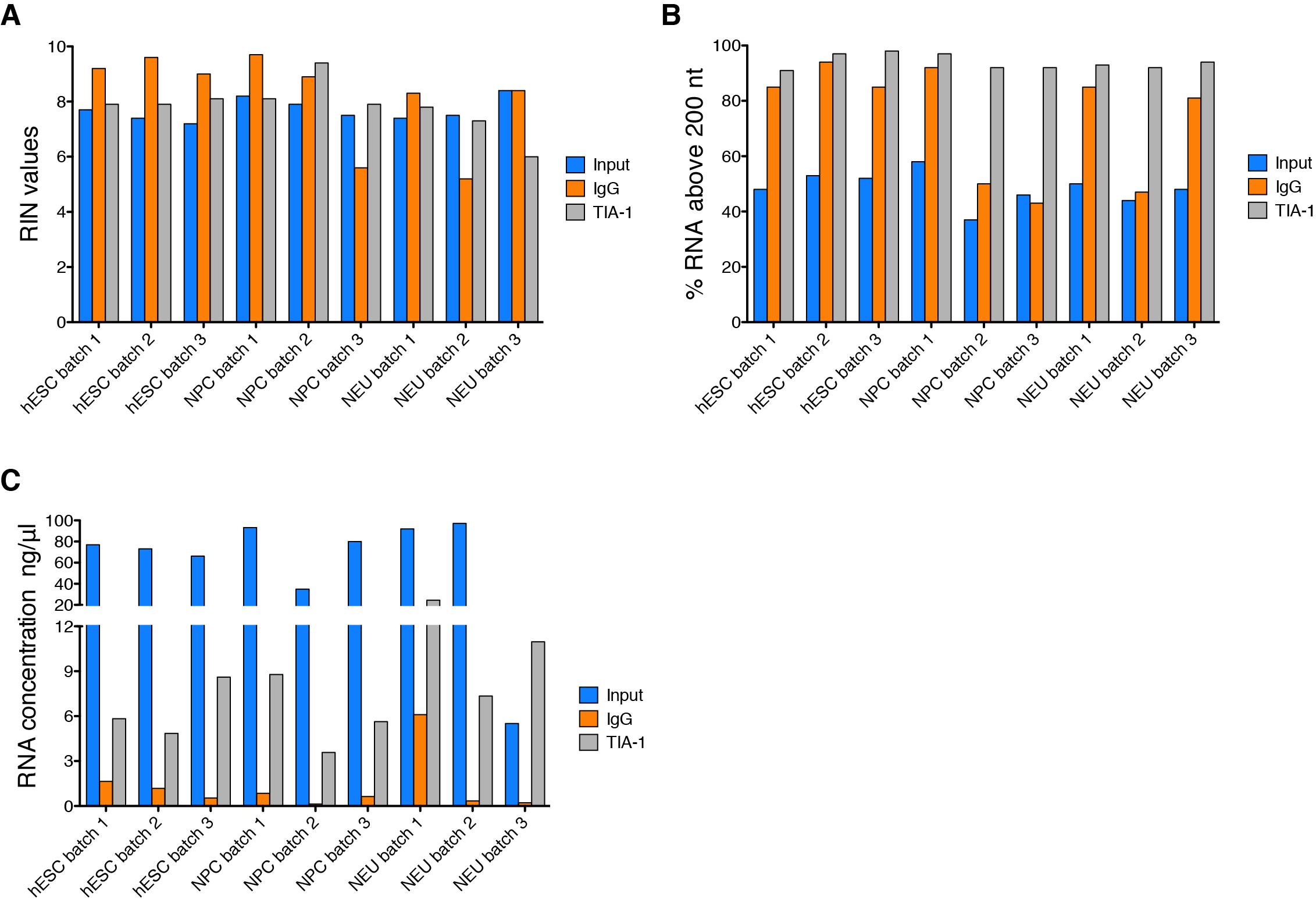

Supplement: Supplementary file 1 — Supplementary Figure 1. Quality control of RNA samples used for sequencing. A RNA Integrity Numbers (RIN) from all RNA samples show that all input samples have RIN values above 7. B percentage of RNA molecules larger than 200 nucleotides for each sample. C RNA concentration of all samples show that IgG samples had low yields in RNA recovery. Supplementary file1 (JPG 494 kb) [file 11033_2021_6634_MOESM1_ESM.jpg]
